# Implementation of active learning methods by nurse educators in undergraduate nursing students’ programs – a group interview

**DOI:** 10.1186/s12912-021-00688-y

**Published:** 2021-09-18

**Authors:** Sanela Pivač, Brigita Skela-Savič, Duška Jović, Mediha Avdić, Sedina Kalender-Smajlović

**Affiliations:** 1 Angela Boškin Faculty of Health Care, Spodnji Plavž 3, SI-4270 Jesenice, Slovenia; 2 Faculty of Medicine, Department of Health Care, University of Banja Luka, Banja Luka, Bosnia and Herzegovina; 3 Public Institution Health Centre of Sarajevo Canton, Sarajevo, Bosnia and Herzegovina

**Keywords:** Nursing education, Teaching tools, Critical thinking, Communication skills

## Abstract

**Background:**

Modern and active learning methods form an important part in the education of Nursing students. They encourage the development of communication and critical thinking skills, and ensure the safe health care of patients. Our aim was to obtain naturalistic data from nurse educators regarding want the use and effects of implementing active learning methods (Peyton’s Four-Step Approach, Mind Mapping, Debriefing and Objective Structured Clinical Examination methods) in the study process of students of Nursing after a completed education module, *Clinical skills of mentors*, as part of the Strengthening Nursing in Bosnia and Herzegovina Project. We wish to learn about the perception of nurse educators regarding the use of active learning methods in the study process of Nursing in the future.

**Methods:**

Qualitative research was conducted and a group interview technique was used for data collection. Beforehand, research participants were included in a two-day education module, *Clinical skills of mentors*, as part of the Strengthening Nursing in Bosnia and Herzegovina Project. Content analysis of the discussion transcriptions was conducted.

**Results:**

Fourteen nurse educators participated. Group interviews were conducted in September 2019. The obtained categories form four topics: (1) positive effect on the development of students’ communication skills (2) positive effect of learning methods on the development of students’ critical thinking skills (3) ensuring a safe learning environment (4) implementation of active learning methods.

**Conclusions:**

The use of various active learning methods in simulation settings improves the Nursing students’ critical thinking and communication skills. Therefore, we believe that Peyton’s Four-Step Approach, Mind Mapping and Debriefing methods should be included as tools for effective student learning and as preparation for directly performing safe nursing interventions with a patient. Effective approaches to the assessment of Nursing students may ensure quality patient health care in accordance with the vision of the nursing profession.

## Introduction

Education of nurses in Bosnia and Herzegovina (BiH) has been undergoing rapid development. In BiH, the emphasis of the health care system and the education of health professionals is mainly on curative care and medical services. This might limit the potential of the nursing staff to react to the population’s present and future health needs. To avoid the negative consequences of reduced competencies and practice, nurses developed a project that would strengthen nursing in BiH [1].

Several nursing-related challenges that currently have a negative effect on the nursing profession and which, therefore, hinder health outcomes in BiH, were identified in collaboration with major stakeholders in BiH, including faculties of nursing and other important organizations [2]. As stated by Francis and O’Brien [3], teaching clinical skills is an important part in educating students of Nursing in an increasingly more complex health care and social environment. As a method of teaching, skills-lab training is viewed as a key component of curricula in the majority of our faculties that offer health-related study programs as it enables a protected training environment where students are permitted to make mistakes and where they can practice their skills on mannequins before working on real patients [4].

In BiH, mentorship at faculties of Nursing is conducted by graduate nurses who are teaching assistants or senior teaching assistants rather than mentors, and are employed full-time or are outsourced. In BiH, there are no additional training options that would equip nurses to work as mentors, either full time or part time, so they only need to meet the general and specific criteria for an associate/teacher position [5]. That is why in 2017, the Strengthening Nursing in BiH Project developed a training program for BiH clinical skills mentors that comprised seven modules (34 h in total) and was focused on different aspects of adult education, and teaching tools and methods. In BiH, the faculties offering Nursing courses have been faced with challenges regarding the organization of additional training for clinical skills mentors. In order for mentors to be prepared for this role as best as they can, they use professional literature, the internet and engage in team meetings [6].

## Background

In order to improve nursing education, various teaching methods have been introduced to assist students in gaining knowledge, skills and attitudes that are relevant for nursing practice.

The implementation of active learning methods into the study process might result in students’ improved motivation for learning, encourage their critical thinking skills and independent learning [7]. Rather than continuing with employing traditional teacher-centered educational approaches, faculties should introduce an active student-centered learning environment since creating learning experiences that encourage reflection, knowledge building, problem-solving, inquiry, and critical thinking are highly significant [8]. Authors [9, 10] state that active learning methods in nursing education are highly significant with an aim to eliminate passive listening and transition to assuming an active role in the educational process and obtain the ability to apply information from lectures in a meaningful way. The development of the pedagogical skills occurs according to the actual situation in the process of the nurse’s practical work [11].

The use of simulation-based learning contributes to the development of students’ sense of safety when they perform various tasks [12]. The skill laboratory functions as a transitional setting between a classroom and clinical venues [13].

Peyton’s Four-Step Approach is a learning method that comprises four steps and is highly effective in the learning process of nursing interventions. The first step is *demonstration*, in which the teacher demonstrates the intervention at their normal pace without giving any additional verbal explanations. The second step is *deconstruction*, in which the teacher performs the intervention by giving detailed descriptions of all the phases of the intervention. In the third step referred to as *comprehension*, the teacher performs the intervention according to the student’s instructions of each step of the intervention. In the final, fourth step called *intervention*, students perform the intervention by themselves without the help of the teacher [14]. In the research on the effectiveness of the method, the authors found that the Peyton’s Four-Step Approach method enables students’ active involvement in the process of learning about nursing intervention [15]. The process of self-explanation, which Peyton’s Four-Step Approach contains when a student is thinking aloud, enables an improved learning process and the development of critical thinking skills [16].

The Mind Mapping method is an excellent pedagogical tool used to help students achieve positive learning outcomes [17] that may be successfully implemented in the education process as it ensures a creative environment and is an effective tool for teachers, mentors, students and researchers [18]. The Mind Mapping method encourages students to obtain relevant information and develops critical thinking skills, which in turn, has positive effects on the provision of safe health care for patients [19]. There are several reasons why using the Mind Mapping method in learning and teaching is recommended. Firstly, there is no long text. Also, it enables learning through synthesizing, as well as clarification and better reorganization of ideas. Furthermore, it assists with revision, encourages visualization of the content that had been learnt before, enables cooperation via studying in groups, which has positive consequences for everybody involved, and finally, mind maps that are submitted to the group result in a better experience because more participants are involved, which produces more ideas and stimulates the use of critical thinking skills [20–22].

The use of simulation-based learning contributes to the development of students’ sense of safety when they perform various tasks [12]. The skill laboratory functions as a transitional setting between a classroom and clinical venues [13].

A guided discussion is also quite significant in teaching Nursing students as it enables very authentic simulations of reality since a mentor asks students to critically evaluate their knowledge and skills that they had demonstrated while performing the scenario. Despite much research conducted on educating with simulation, the guided discussion has not yet been sufficiently defined [23]. The use of scenarios with debriefing constitutes a strategy facilitating the teaching-learning process in the undergraduate nursing course [24].

The assessment of clinical skills is also highly significant in nursing education. Therefore, the Objective Structured Clinical Examination (OSCE) may be considered to be a sound assessment tool whose objective is the assessment of nursing students’ clinical competences in a safe and controlled environment, which enables simple assessment of the knowledge and performance of clinical skills that are important in nursing practice. Also, the assessment tool may serve to better prepare students for their profession [25, 12].

A higher education teacher is one of the key factors for a nursing student to be successful in their studies [26], so teachers should be familiar with effective teaching methods [27]. A teacher’s primary task is to ensure a creative environment and a learning path that engages a student [28]. A suitable learning method may encourage nursing students to learn, improve students’ communication, and motivate and inform them about effective learning [29].

### Aim of the study

After conducting the educational module *Clinical skills of mentors* as part of the Strengthening Nursing in BiH Project, our aim was to obtain naturalistic data from nurse educators regarding the use and effects of introducing active learning methods (Peyton’s Four-Step Approach, Mind Mapping, Debriefing and Objective Structured Clinical Examination methods) in the study process of Nursing students. Nurse educators in BiH have not yet developed knowledge in the field of active and contemporary learning methods or competencies to transition to modern teaching methods from more traditional ones. That is why we conducted the research to learn about the perception of nurse educators about the use of active learning methods in the study process of Nursing in the future.

### Our research question was

What is the perception of nurse educators on the use and effects of active learning methods in nursing undergraduate study programs?

## Methods

### Design

The qualitative descriptive study of a formal group interview was used [30]. The group interview was conducted in pre-existing “natural” groups of nursing educators in a region of BiH. Invited participants attended a two-day education module: *Clinical skills of mentors* as part of the Strengthening Nursing in BiH Project that was hosted by two higher education senior lecturers of Nursing from Slovenia, where higher education and clinical mentorship in Nursing is developed in accordance with international [31, 32] and national guidelines for Nursing education [33].

A group interview allows group members to influence each other with their comments and experience, and to form an opinion regarding the issue currently discussed after reflecting on it as a group [34], which results in obtaining more information than with face-to-face interviews [35].

### Setting and participants

The environment in which the research was conducted impacted the format of the group [27] that was invited to participate for research purposes. 14 nurse educators that are employed at the faculty (n = 6) and/or in a clinical setting (n = 8) participated in a group interview. 12 women and two men participated, the average age of the participants was 35.7 years (SD = 0.7), the average length of service was 15.1 years (SD = 11.1). One participant had completed doctoral studies, nine held master’s degrees in Nursing and four held bachelor degree in Nursing.

### Instrument

Semi-structured guiding questions that were being updated throughout the discussion were used in the group interview. The semi-structured interview contained eight basic questions:

- What is your opinion regarding the significance of the qualifications of nurse educators to work with Nursing students?
- What skills should nurse educators have in order to teach students of Nursing effectively?
- How useful do you think the Peyton’s Four-Step Approach method is for teaching Nursing students?
- What is your opinion on the usefulness of the OSCE stations as a method of assessing Nursing students?
- How do you assess the usefulness of the Mind Mapping method in the learning process?
- What is your attitude towards using and implementing the Debriefing method in the process?
- Which presented method do you think is most suitable for teaching the students of Nursing?
- What is your attitude towards the impact of the presented learning methods and assessment on the development of critical thinking skills of Nursing students?

The reliability of the research was ensured by considering the homogeneity of the group, an appropriate number of participants in the interview, by giving instructions before the start of the interview emphasizing that everyone has the possibility to participate in the discussion and by encouraging them with questions when guiding the group interview [36]. The reliability check was ensured by digitally recording the conversations in the group interview. We then transcribed the recording and checked that the transcription matched the audio recording. To increase the validity of data analysis, two researchers analyzed the data and ensured that the codes were unified. An independent analysis of two researchers decreases the possibility of partiality and increases the interpretative basis of the research. With the thematic analysis, we demonstrated that data analysis has been conducted in a precise, consistent, and exhaustive manner through recording, systematizing, and disclosing the methods of analysis with enough detail [37]. Also, we used traditional tools: colored pens, paper, and sticky notes for ensuring rigor [38]. Authors checked the participants to ensure relevant evaluation [39].

### Data collection

The group interview took place in September 2019 at the end of the education module and was guided by a moderator and a semi-structured questionnaire. There was also an administrator who recorded the discussions. The research participants attended a two-day education module, *Clinical skills of mentors*, as part of the Strengthening BiH Project. The purpose of the module was to inform and educate nurse educators about active learning methods and assessment of students of Nursing: Mind Mapping, Debriefing, Peyton’s Four-Step Approach methodology and the OSCE stations. We presented the purpose of the research and topics before conducting the group interview. The average time of the group interview was 90 min. Discussions were recorded upon obtaining a written permission by the participants. To ensure anonymity we added randomly selected letters to the interviewers.

### Data analysis

For qualitative data, the method of thematic content analysis was employed. All recordings were transcribed verbatim and the texts were read several times. After coding units were identified, coding was conducted and categories and key topics were defined. Each participant in the group interview was ascribed a corresponding code. The nominal identity of a transcription was lost while the traceability of content was ensured. Authors state that a thematic analysis is a widely used qualitative analytical method that offers an accessible and theoretically adjustable approach to the analysis of qualitative data [40].

## Results

Based on an analysis of the text, 47 codes were designed alongside 14 umbrella categories. The obtained categories fall into four final topics: (1) a positive effect on the development of students’ critical thinking skills (2) a positive effect of learning methods on the development of students’ critical thinking skills (3) providing a safe learning environment (4) implementation of active learning methods (Table 1).

**Table.1:** The results of the discussion on the use of active learning methods in Nursing undergraduate study programs

Categories	Number of codes	Topics
Communication skills	5	Positive effect on the development of students’ communication skills
Feedback	4	
Memorizing	1	
Encouraging critical thinking	8	Positive effect of learning methods on the development of students’ critical thinking skills
Reflection of students on the issue	1	
Problem solving	1	
Active role of students	3	
Safety	5	Ensuring a safe learning environment
Significance of learning strategies for patients	3	
Students’ perspective	5	
Learning with simulations	4	
Assessment approaches	3	
Usefulness of methods	2	Implementation of active learning methods
The roles and skills of teachers	2	

**Table.2:** Effects of active learning methods in Nursing study programs, and suggestions based on the discussion with nurse educators

Topics	Selected examples of comments	Effects	Obstacles consequences	Suggestions
Positive effect on the development of students’ communication skills	*“I would like to stress that communication is very important between students and mentors…which means that mentors pass on their knowledge and experience through communication.”* *(Person K)* *“…. Communication is very important and also our thinking, we should teach our students through role play.” (Person F)* *“…with communication we can solve a problem.” (Person I)* *“It is important that a student gets information immediately.” (Person D)* *“A teacher’s feedback is very important, so that the student knows where they were wrong and what they should do to improve.” (Person B)*	Achieving professional competencies in communication. Decreasing errors in patient care. Transfer of knowledge and experiences to students of Nursing. The impact of feedback on the student’s active role and achieving set goals. Teaching by including active problem solving that leads students to reflect and encourages them to show and say what they know. Assessment of the health condition. Prevention of errors in a clinical setting. Active participation of all students. Development of self-confidence in students. Students are better motivated.	Feedback by nurse educators should be useful and given at the right time as, otherwise, it is not useful. It should be given when a student is reflecting on their work and there is still time for improvement. The OSCE method requires appropriate logistics and extra support (time, place).	With learning and direct work a student progresses from an inexperienced individual to a more independent, professional and responsible person, so strengthening students’ interpersonal communication competencies is necessary: communication skills, a positive and professional attitude, active listening, the ability to reflect and respond emphatically, constructive feedback that can be achieved by introducing active learning methods into the study process. Nurse educators should adjust the teaching methods depending on the resources, knowledge, experience of students and situation.
Positive effect of learning methods on the development of students’ critical thinking	*“A student should definitely be encouraged to think and connect all the facts that they have through the Peyton’s four-step approach method.” (Person I)* *“It is great for the development of critical thinking.” (Person F)* *“It means that they will know what is best for the patient” (Person G)* *“With the Debriefing method we will make students think critically and so they will know what they have missed….it is important to have a discussion with students.” (Person B)* *“Through Mind Mapping students and those students who usually don’t speak as much, will participate in discussions.” (Person B)* *“The disadvantage of the OSCE method is the venue that cannot cater for that many stations, but otherwise, it is a great method for assessment.” (Person E)*			
Ensuring a safe learning environment	*“The main goal is to ensure the safety of our patients”. (Person I)* *“….the Peyton’s four-step approach can also be used for education of patients, which means that it has a large spectrum”. (Person K)*	Preparation for safe nursing intervention. Safe and high-quality patient care in a clinical setting.	Peyton’s four-step approach requires following in steps.	To ensure a systematic approach to improve the quality and safety of everyone involved.
Implementation of active learning methods	*“I’d like to add that teachers should have pedagogical and andragogical knowledge and skills and mentoring skills.” (Person J)* *“During the OSCE exam it is easier to follow two people and that they then make comparisons” (Person Y)* *“… that these simulations are necessary and that in this way we can prepare students for their work well, but that for that we need to have knowledge.” (Person I).* *“In my work I will use the OSCE stations because I think that this is a good way to check students’ resourcefulness in addition to their knowledge and skills.” (Person C)* *“Using the Mind Mapping method may be the cheapest method and does not take a lot of time, but is very effective for the students.” (Person B)*	Standardized methods with precisely defined criteria and phases of performing clinical interventions. Improvement of knowledge and clinical interventions. Preparation for work with a patient is very important.	Lack of knowledge of nurse educators on active learning methods. Nurse educators do not have enough pedagogical and andragogical knowledge.	Strengthening nurse educators with knowledge in pedagogy and andragogy, and active teaching methods.

Table 2 presents topics that are substantiated by representative quotes, their effects on the education of students of nursing, the obstacles and consequences that nurse educators have observed, and suggestions regarding improvements based on the presented topics and statements.

### 1. A positive effect on the development of students’ communication skills

 Participants in the group interview believed that various active methods have a positive effect on the development of Nursing students’ communication skills, and on achieving professional competencies in communication. They emphasize the importance of feedback exchanged between a student and a nurse educator that should be given at a time when a student is still thinking about their work and there is still time to improve the process itself. They think that feedback has an impact on the active role of a student and on achievement of the set goals. The research participants believe that showing nursing interventions several times during the learning process has positive effects on students’ memorizing skills and results in less errors when providing health care to patients.

### 2. The effect of learning methods on the development of critical thinking

In their statements, the participants of the group interview most often stated that using various innovative methods in a simulation setting encourages critical thinking skills of nursing students, impacts the development of students’ self-confidence and motivates them to work and study. Active participation and critical thinking lead students to successful problem solving. Participants believe that teaching by means of actively solving problems results in students’ reflecting and encourages them to show and say what they know. The participants emphasize that using certain methods of learning also depends on external resources.

### 3. Ensuring a safe learning environment

The participants of the group interview all thought that methods such as the Peyton’s Four-Step Approach method, the OSCE stations and Debriefing contribute to a safe clinical environment since students revise their knowledge and skills several times in simulated conditions and in this way strengthen their knowledge. They believe that preparing students to perform safe nursing intervention is connected to safe and quality patient health care in a clinical setting.

### 4. Implementation of active learning methods

The nurse educators included in the research found the presented methods highly suitable for use in the education process of Nursing students, but emphasized that a suitable venue should be provided, and that teachers or clinical mentors should be suitably prepared. They think that nurse educators neither have enough pedagogical and andragogical experiences and skills, nor knowledge on active learning methods.

## Discussion

With qualitative research, we obtained the views and opinions of nurse educators from some nursing faculties and clinical settings in BiH on the effects of active learning methods on students of nursing. Participants in the group interview believed that various active methods have a positive effect on the development of Nursing students’ communication skills, and on achieving professional competencies in communication. They emphasized the importance of feedback exchanged between a student and a nurse educator. Other research [41–43] also emphasizes the importance of communication skills, giving feedback and providing quality in nursing care. In our study the participants of the conducted research thought that active learning methods contribute to a more effective learning of nursing interventions, provide an active approach to learning about nursing interventions and enable a more confident performance of nursing interventions in a clinical setting with a patient, and provide a safe environment either for the patient or for the student.

Participants believe that the use of innovative methods in a simulation environment encourages students’ critical thinking, motivation, development of self-confidence and problem-based learning. Christianson Krista [44] puts great importance on the connection between emotional intelligence and critical thinking in nursing education. A study [45] demonstrated that critical thinking education improves problem-solving skills. A good relationship between a nurse educator and a student brings positive results in students’ education and motivates them for work in a clinical setting [46]. In addition, a teacher’s professional knowledge and their organizational and communication skills also have an effect on successful learning [47]. Baksi et al. [48] researched the effects that clinical preparatory education provided before the first clinical experience had on anxiety.

 The opinions of the participants regarding the Debriefing method mostly refer to the importance of feedback. In researching debriefing practices in interprofessional simulation with students from the socio-material perspective, the findings have shown how debriefing intertwines with, and is shaped by social and material relationships. Two patterns of enacting debriefing were identified. First, debriefing as an algorithm was enacted as a protocol-based, closed inquiry approach and secondly, laissez-faire debriefing was enacted as a collegial conversation with an open inquiry approach that has a loose structure [49].

Nursing educators believe that active pedagogical methods of learning might help students to optimize the development of their critical thinking skills. Therefore, we believe that Peyton’s Four-Step Approach method, Mind Mapping and Debriefing should be included as a tool for successful learning of students and preparation for working directly with patients, which has already been pointed out by previous research conducted by Janicas and Narchi [24] and Francis and O’Brien [3].

The use of Mind Mapping helps students to understand their thinking process, obtain basic knowledge that they will upgrade with in-depth professional knowledge [18], and encourages them to learn independently, be independent and think critically [50]. While students were developing mind maps, they were also exploring the critical thinking concept by reflecting on how they make patient care decisions in a clinical setting [22]. The reflection resulted in the students being better able to describe their critical thinking process and demonstrate the concept graphically. Mind Mapping may be used to illustrate the pathways that encourage reflection on patient care [51].

The participants of our research believe that the methods that were presented and demonstrated with examples contribute to a safe clinical setting. Clark [25] also emphasizes that one of the more challenging tasks of faculties of health sciences is to educate students with competencies that will secure safe and effective nursing and patient care. In the research, nurse educators believe that active methods are very useful in the education process of Nursing students, but emphasize that an appropriate location should be provided and that nurse educators should be suitably trained. They also believe that nurse educators must have pedagogical and andragogical knowledge, so that they can implement active and quality teaching methods in the pedagogical process. The challenges in the training of nursing educators are in the transition from traditional education to simulated learning environment [52]. Addressing nurse educator challenges and empowering them with the means, opportunity and skills to utilize student-centered teaching and learning strategies may contribute to the development of undergraduate student nurses’ clinical reasoning skills. Raising awareness of the challenges that nurse educators experience in implementing student-centered facilitation of learning can assist in developing the strategies to ensure nurse educators become more student-centered in their teaching [53].

Based on the conducted research, we propose an implementation of active teaching methods in the educational process of nursing students. Recommendations relate to regular monitoring of learning objectives, evaluation, updating teaching methods and the transition from traditional forms of learning to innovative forms.

## Limitations and Strengths

Nurse educators that had previously been included in the training on active teaching methods in Nursing were included in the research. An advantage of the research is that we have obtained the views and opinions of nurse educators regarding the usefulness and effectiveness of the presented methods in the undergraduate study program of Nursing immediately after the conducted theoretical and practical training. An important limitation is also the local context of nursing development in BiH. The convenient sampling does not always allow us to select the best representatives related to the research question. It is likely that the members included in the group are more motivated for improvements in nursing education than other nurses at faculties and clinical settings.

Further research should focus on two areas. Firstly, it should be researched how often active learning methods are used in the education process and secondly, the opinions of students on how active learning methods impact the understanding and learning of nursing intervention could be obtained. The limitation of the research is the time span of the education on active teaching methods and the selection of guiding questions, since we are aware that the selection of questions might partially impact the answers given by the participants. It may be supposed that a different selection of guiding questions might result in different opinions of nurse educators.

## Conclusions

The results of the qualitative research were used to present the meaning and understanding of implementing active learning methods in undergraduate study programs of Nursing. Based on the conducted research we believe that nurse educators lack pedagogical and andragogical knowledge as well as knowledge of active learning methods. The use of active learning methods by nurse educators improves communication skills and develop students’ critical thinking skills. The final results of implementing active learning methods are in the acquired learning outcomes, nursing interventions that are performed optimally, development of competent professionals, boosting self-confidence, improvement of communication and critical thinking skills, as well as the self-initiative and readiness to work with patients in a clinical setting. Cutting edge and active learning methods are important for ensuring the quality of a study process and in the teaching of students of Nursing. We also wish to encourage study programs where the presented learning methods would be used in the study process, so that students will become active participants who can use their knowledge in providing safe patient care as a result of these active methods of teaching and learning.

We considered ethical principles of ensuring the voluntary nature of participating in the research, protecting the identity of individuals, confidentiality, privacy and respecting the truth. Research was carried out in accordance with the Helsinki-Tokyo Declaration [54], the Code of Ethics for Nurses and Nurse Assistants of Slovenia [55] and the ethical guidelines of the Social Research Ethics Guidance [56, 57]. We did not apply for the approval by the National Medical Ethics Committee as the National Medical Ethics Committee only makes decisions concerning experimental research and non-experimental research conducted on patients and vulnerable groups in the population (patients, the elderly, children, disabled adults). Nurse educators do not form part of vulnerable groups and our questions were not considered to belong to the group of vulnerable questions. The study is exempt from ethical approval according national legislation (European Commission: Accession countries - legislation related to research ethics Slovenia).

## Data Availability

The datasets used and/or analyzed during the current study are available from the corresponding author on reasonable request.

## Abbreviations

BiH: Bosnia and Herzegovina
OSCE: Objective Structured Clinical Examination
